# Design, synthesis and cytotoxic effects of curcuminoids on HeLa, K562, MCF-7 and MDA-MB-231 cancer cell lines

**DOI:** 10.1186/s13065-018-0398-1

**Published:** 2018-03-19

**Authors:** Siti Noor Hajar Zamrus, Muhammad Nadeem Akhtar, Swee Keong Yeap, Ching Kheng Quah, Wan-Sin Loh, Noorjahan Banu Alitheen, Seema Zareen, Saiful Nizam Tajuddin, Yazmin Hussin, Syed Adnan Ali Shah

**Affiliations:** 1 Faculty of Industrial Sciences & Technology, Universiti Malaysia Pahang, Lebuhraya Tun Razak, 26300 Gambang Kuantan, Pahang Malaysia; 2 Bio-Aromatic Research Center of Excellence, Faculty of Industrial Sciences & Technology, Universiti Malaysia Pahang, Lebuhraya Tun Razak, 26300 Gambang Kuantan, Pahang Malaysia; 3 China-ASEAN College of Marine Sciences, Xiamen University Malaysia, Jalan Sunsuria, Bandar Sunsuria, 43900 Sepang, Selangor Darul Ehsan Malaysia; 4 X-ray Crystallography Unit, School of Physics, Universiti Sains Malaysia, 11800 USM Pulau, Pinang Malaysia; 5 Department of Cell and Molecular Biology, Faculty of Biotechnology and Biomolecular Science, Universiti Putra Malaysia, 43400 Serdang, Selangor Darul Ehsan Malaysia; 6 Research Institute of Natural Products for Drug Discovery (RiND), NMR Facility Division, Faculty of Pharmacy, Universiti Teknologi MARA (UiTM), Puncak Alam Campus, 42300 Bandar Puncak Alam, Selangor Darul Ehsan Malaysia

**Keywords:** Curcuminoids synthesis, Breast cancer cell lines, SARs, (2*E*, 6*E*)-2, 6-bis(2- methoxybenzylidene)cyclohexanone

## Abstract

**Background:**

Curcumin is one of the leading compound extracted from the dry powder of *Curcuma longa* (Zingiberaceae family), which possess several pharmacological properties. However, in vivo administration exhibited limited applications in cancer therapies.

**Results:**

Twenty-four curcumin derivatives have synthesized, which comprises cyclohexanone **1**–**10**, acetone **11**–**17** and cyclopentanone **18**–**24** series. All the curcuminoids were synthesized by the acid or base catalyzed Claisen Schmidt condenstion reactions, in which β-diketone moiety of curcumin was modified with mono-ketone. These curcuminoids **1**–**24** were screened against HeLa, K562, MCF-7 (an estrogen-dependent) and MDA-MB-231 (an estrogen-independent) cancer cell lines. Among them, acetone series **11**–**17** were found to be more selective and potential cytotoxic agents. The compound **14** was exhibited (IC_50_ = 3.02 ± 1.20 and 1.52 ± 0.60 µg/mL) against MCF-7 and MDA-MB-231 breast cancer cell lines. Among the cyclohexanone series, the compound **4** exhibited (IC_50_ = 11.04 ± 2.80, 6.50 ± 01.80, 8.70 ± 3.10 and 2.30 ± 1.60 µg/mL) potential cytotoxicity against four proposed cancer cell lines, respectively. All the curcucminoids were characterized with the detailed ^1^H NMR, IR, UV–Vis, and mass spectroscopic techniques. The structure of compound **4** was confirmed by using the single X-ray crystallography. Additionally, we are going to report the first time spectral data of (2*E*,6*E*)-2,6-bis(2-methoxybenzylidene)cyclohexanone (**1**). Structure–activity relationships revealed that the mono-carbonyl with 2,5-dimethoxy substituted curcuminoids could be an essential for the future drugs against cancer diseases.

**Conclusions:**

Curcuminoids with diferuloyl(4-hydroxy-3-methoxycinnamoyl) moiety with mono carbonyl exhibiting potential cytotoxic properties. The compound **14** was exhibited (IC_50_ = 3.02 ± 1.20 and 1.52 ± 0.60 µg/mL) against MCF-7 and MDA-MB-231 breast cancer cell lines.
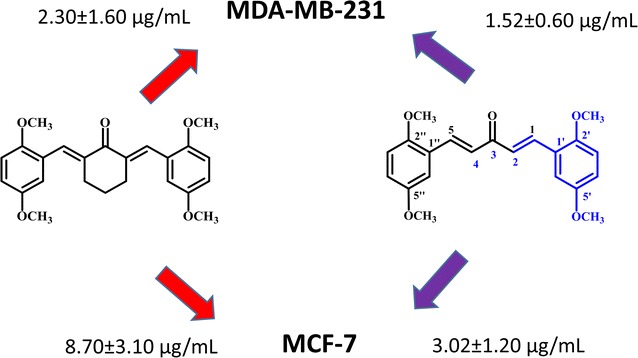

## Introduction

Cancer is one of the leading causes of death worldwide, with approximately 14 million new cases in 2012 [1]. The number of new cases is expected to rise by about 70% over the next two decades. Cancer causes of death globally and was responsible for 8.8 million deaths in 2015. Globally, nearly 1 in 6 deaths is due to cancer [2]. In 2016, 1,685,210 new cancer cases and 595,690 cancer deaths are projected to occur in the United States [3]. Breast cancer was the commonest cancer in women amongst all races from the age of 20 years in Malaysia for 2003 to 2005. According to the National Cancer Institute, 232,340 female breast cancers and 2240 male breast cancers are reported in the USA. It accounts for 16% of all female cancers and 22.9% of invasive cancers in women [4–6]. Curcumin (1,7-bis-(4-hydroxy-3-methoxyphenyl)-1,6-heptadiene-3,5-dione) is a natural diarylheptanoid extracted from the rhizome of *Curcuma longa* [7, 8]. Curcumin is a fascinating symmetrical molecules possessing interesting skeleton of β-diketone with diferuloyl (4-hydroxy-3-methoxycinnamic acid) moieties [9]. It exhibited remarkable biological activities mainly anticancer [10–12], anti-inflammatory [13–15], antioxidant [16, 17], anti-hepatotoxic [18], nephroprotective [19], thrombosis suppressing [20], and hypoglycemic activities [21]. Curcuminoids have been identified as a potent anti-breast cancer agent available from natural food ingredients including turmeric. This effect maybe contributed through targeting the estrogen receptors [22]. Advance understanding of bioactive metabolites through chemical synthesis has further enhanced the potential of these natural products including curcumin as the anticancer agent. For example, 4-hydroxy-3-methoxybenzylidene)-*N*-methyl-4-piperidone (PAC), which is the analogue of curcumin were reported with enhanced antitumor effect against breast cancer via targeting the estrogen receptor [23]. On the other hand, modification of cyclohexanone derivative of curcumin was reported to enhance cytotoxicity against estrogen receptor-negative breast cancer cells [24]. Although it is well known natural remedies for pain still have bioavailability problems such as absorption, distribution, metabolism etc. [25, 26]. Due to its significant anti-cancer properties on the various cancers such as gastrointestinal, genitourinary, gynecological, hematological, pulmonary, breast, and bone diseases, curcumin becomes a promising lead compound to develop a novel drugs [27, 28].

## Results and discussion

### Chemistry

Curcuminoids are the derivatives of curcumin. About **24** curcuminoids have been synthesized and investigated their cytotoxic properties against various cancer lines and thus established the structure–activity relationship for the future drugs development. In our experiments, we have synthesized three series of mono-carbonyl analogues of curcuminoids with cyclohexanone (**1**–**10**), acetone (**11**–**17**) and cyclopentanone (**18**–**24**). Three series were synthesized by Claisen–Schmidt condensation reaction by coupling the appropriate aromatic aldehydes with cyclohexanone, acetone and cyclopentanone by acid or base catalysed as previously stated by Wei [29]. In this project, β-diketone moiety of curcumin was modified with mono ketone and investigated their cytotoxic properties against Hele cell lines (human cervical cancer), K562 (Leukemia) cell lines, MCF-7 (an estrogen-dependent) and MDA-MB-231 (an estrogen-independent) cancer cell lines [30]. Additionally, we are going to report first time the data of (2*E*, 6*E*)-2,6-bis(2-methoxybenzylidene)cyclohexanone (**1**). Recently, we have reported the in vivo anti-tumour activity of 2,6-bis(4-hydroxy-3-methoxybenzylidene)cyclohexanone (**5**) on 4T1 breast cancer cells [31]. Previously, we have published another curcumin derivative DK1 and naturally occurring chalcone flavokawain B and its derivatives on various breast cancer cell lines [32–34].

**Fig. 1:**
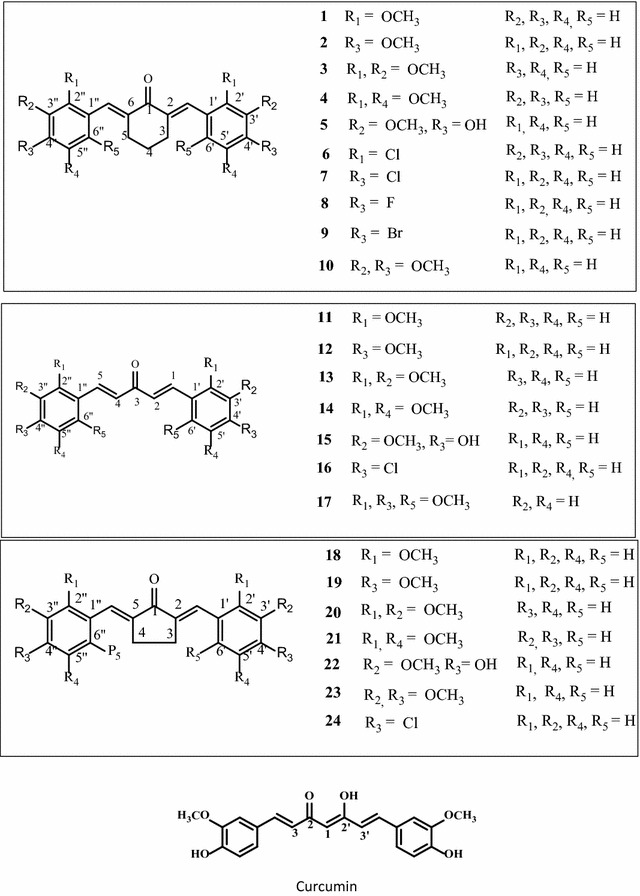
Chemical structures of curcuminoids (**1**–**24**) and curcumin

The compound was **1** purified as yellow liquid. The UV spectrum of compound **1** showed the absorption wavelength, λ_max_ at 339 nm corresponding to the α,β conjugated carbonyl group (C=O) compound. The IR absorption bands at 1636 cm^−1^ corresponding to carbonyl (C=O) and 2942–3001 cm^−1^ referred to aromatic C–H stretching functional groups. The ^1^H NMR spectrum (600 MHz, CDCl_3_) of compound **1** appeared at δ 1.75 as multiplet (2H) was assigned to the methylene proton (CH_2_) at C4. A methylene protons at 2.84 as a multiplet (4H) integrated was corresponding to the C3 and C5 atoms. A singlet appeared at 3.86 integrated by 6H was assigned to the methoxy protons (OCH_3_) at C2′ and C2″ position. A multiplet appeared at 6.92 was assigned to the aromatic protons at C3′ and C3″ methine protons. Two protons (2H) integrated at 6.96 shown a multiplet were assigned to the C5′ and C5″ protons. Another multiplet appeared at 7.33–730 (4H) was assigned to the C4′, C4″, C6′ and C6″ as aromatic methine protons. A broad singlet appeared at 7.98 integrated by 2H was due to the olefinic protons (–C=C–H). The board band decoupled spectra ^13^C NMR showed the presence seven quaternary carbons, three methylene and ten methine carbons atoms. The compound showed EI-MS molecular mass was at *m/z* 334. The molecular formula of compound **1** was supported by HREI-MS calculated C_22_H_22_O_3_ 334.1575, found for 334.1580, which supported the proposed structure of compound **1** (Fig. 1). Previously, the radical scavenger and enzyme inducer activity of compound **1** obtained from Aldrich was reported by Dinkova-Kostavo et al. [35]. Interestingly, the data of all the compounds were characterized precisely on 600 MHz Bruker and 500 MHz and assignments were made carefully. The data of known compounds were compared with the previously published by Wei, Hosoya and Du [29, 36, 37].

### Structure–activity relationship

All the curcuminoids have been screened against HeLa, K562, MCF-7 and MDA-MB-231 cancer cell lines and results are depicted in Table 1. Among the cyclohexanone series **1**–**10**, compound **4** was the most potent cytotoxic against four cancer lines especially breast can cell lines exhibited (IC_50_ = 11.04 ± 2.80, 6.50 ± 01.80, 8.70 ± 3.10 and 2.30 ± 1.60 µg/mL), respectively. Compound **5** possess the partial structure of curcumin showed (IC_50_ = 6.03 ± 1.70 and 3.03 ± 1.00 µg/mL) against MCF-7 and MDA-MB-231 breast cancer cell lines almost three to four times more active than curcumin (Table 1). Others curcuminoids **1**, **3**, **6**, **7**, **8**, **10** also showing good cytotoxicity against breast cancer lines MCF-7 and MDA-MB-231 and moderated against HeLa and K562 cell lines. Curcuminoids with acetone series **11**–**17** exhibited more potential cytotoxic effects on four type cancers cell lines, which is comparable with curcumin IC_50_ values in Table 1. Among acetone series, the compound **14** was found to be the most cytotoxic in breast cancer lines MCF-7 and MDA-MB-231 and moderate against HeLa and K562 cell lines. Compound **11** also exhibited (IC_50_ = 11.31 ± 1.33, 4.50 ± 1.20 and 2.07 ± 1.75 µg/mL) against HeLa, MCF-7 and MDA-MB-231, respectively. Other curcuminoids **15**, **16**, and **17** possessing Cl, Br and F substituted showing moderate cytotoxicity against four cancer lines (Table 1). Compound **17** with trimethoxy substituted also exhibiting potential cytotoxicity with (IC_50_ = 2.50 ± 1.10 and 3.10 ± 1.06 µg/mL) against breast cancer lines MCF-7 and MDA-MB-231, which is compatible with the previously published by Fuchs [38]. Curcuminoids **18**–**24** with cyclopentanone series did not show any significant cytotoxicity against all types of cancer lines except compound **22**, showing better cytotoxic effects against Hela and MCF-7 and MDA-MB-231 cancer then curcumin. The lower cytotoxicity of compounds **18**–**24** possibly due to the ring strain, which could be sterically not well-fitted with the estrogen receptors. Cytotoxic results of curcuminoids with acetone series **1**–**10** and methoxy substituted exhibiting selectively more potential than cyclohexanone (**11**–**17**) and cyclopentanone (**18**–**24**) series. The results are summarized in Table 1.

**Table 1:** IC_50_ values of curcuminoids against HeLa, K562, MCF-7 and MBA-MB-231

Compounds	HeLa	K562	MCF-7	MDA-MB-231
	IC_50_ (µg/mL)			
**1**	9.21 ± 1.20	16.04 ± 1.30	3.46 ± 1.22	3.01 ± 0.60
**2**	38.03 ± 3.10	30.12 ± 3.30	42.00 ± 4.20	65.00 ± 4.10
**3**	12.50 ± 1.30	22.50 ± 3.20	8.50 ± 1.50	9.50 ± 1.40
**4**	11.04 ± 2.80	6.50 ± 01.80	8.70 ± 3.10	2.30 ± 1.60
**5**	> 30	17.50 ± 0.50	6.03 ± 1.70	3.03 ± 1.00
**6**	15.07 ± 1.60	20.04 ± 1.10	> 30	> 30
**7**	12.00 ± 1.60	22.50 ± 1.10	10.50 ± 2.10	7.401 ± 1.10
**8**	> 30	> 30	6.50 ± 2.70	3.02 ± 1.10
**9**	11.01 ± 2.10	55.02 ± 3.40	10.50 ± 1.80	6.30 ± 1.30
**10**	> 30	> 30	14.02 ± 1.80	11.90 ± 3.10
**11**	11.31 ± 1.33	15.03 ± 1.90	4.50 ± 1.20	2.07 ± 1.75
**12**	12.01 ± 1.10	32.50 ± 2.10	20.50 ± 2.50	11.00 ± 2.10
**13**	15.20 ± 1.20	> 30	10.00 ± 2.10	9.50 ± 1.10
**14**	14.03 ± 1.40	> 30	3.02 ± 1.20	1.52 ± 0.60
**15**	> 30	15.01 ± 1.30	7.50 ± 1.10	9.20 ± 0.80
**16**	11.00 ± 1.20	12.50 ± 0.95	25.00 ± 3.20	14.21 ± 2.10
**17**	6.15 ± 1.20	> 30	2.50 ± 1.10	3.10 ± 1.06
**18**	> 30	> 30	> 30	18.13 ± 6.10
**19**	> 30	> 30	> 30	> 30
**20**	> 30	> 30	> 30	> 30
**21**	> 30	> 30	> 30	27.50 ± 4.40
**22**	9.00 ± 1.60	> 30	12.50 ± 2.10	6.40 ± 1.10
**23**	> 30	> 30	> 30	> 30
**24**	> 30	> 30	> 30	> 30
**Curcumin**	> 30	> 30	22.50 ± 5.50	26.50 ± 1.40
**Doxorubicin**	4.01 ± 1.20	1.23 ± 1.10	2.50 ± 1.10	0.60 ± 1.10

Data are expressed in terms of ± SE of three independent experiments

Most of curcuminoids are potent as compared to the curcumin with (IC_50_ = 22.50 ± 5.50 and 26.50 ± 1.40 µg/mL) against MCF-7 and MDA-MB-231 (Table 1). Several reports on curcuminoids with mono-carbonyl (acetone series) have been even better pharmacological properties than curcumin [22, 38]. Due to enolization and chelating (hydrogen bonding with the diketone), curcumin exhibited slightly lower cytotoxic effect than the modified derivatives. This could be due to the weak binding with the receptors, thus cause the weak pharmacokinetic profiles [39]. All curcuminoids possessed bis-enone conjugated system, which is quite reasonable site to binding with the Michael receptor selectivity with target nucleophile [30, 40–42]. The curcuminoids with mono-carbonyl **1**–**10** could be potential analogues for the drug discovery against cancer. In this respect, curcumin derivatives bearing a mono-carbonyl and methoxy groups especially cyclohexanone (**1**–**10**) and acetone **11**–**17** series could be a remarkable approach for the improvement of bioavailability problems related to curcumin [43, 44].

### X-ray structure description

**Fig. 2:**
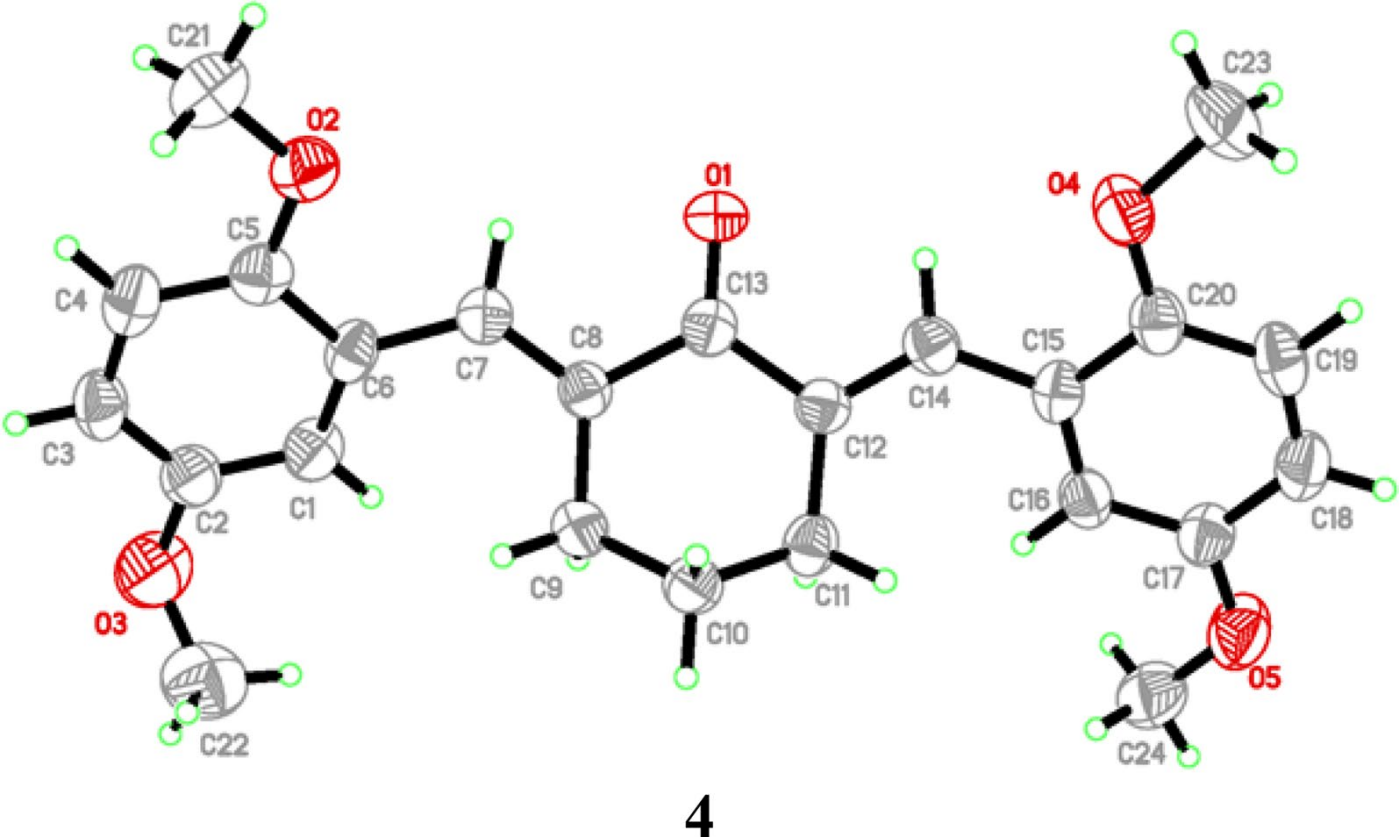
Molecular structures of compound **4** showing the atomic numbering scheme

Crystal data of compound **4** was given in Table 2. One crystal structure was determined by using X-ray diffraction method. Figure 2 showed the molecular structure of compound **4**. Compound **4** crystalized in orthorhombic crystal system, space group *Pna*2_1_.

**Table 2:** Crystal data and parameters for structure refinement of **4**

Crystal data	**4**
CCDC	1548735
Chemical formula	C_24_H_26_O_5_
*M*_r_	394.45
Crystal system, space group	Orthorhombic, *Pna*2_1_
Temperature (K)	296
*a*, *b*, *c* (Å)	8.529 (8), 25.65 (2), 9.430 (8)
α, β, γ (°)	90, 90, 90
*V* (Å^3^)	2063 (3)
*Z*	4
Radiation type	Mo Kα
µ (mm^−1^)	0.09
Crystal size (mm)	0.47 × 0.24 × 0.05
Data collection	
Diffractometer	Bruker APEXII DUO CCD area-detector diffractometer
Absorption correction	Multi-scan (*SADABS*; Bruker, 2009)
*T*_min_, *T*_max_	0.8434, 0.9624
No. of measured, independent and observed $\left[ {{\text{I}}\,{ > }\, 2\sigma \left( I \right)} \right]$ reflections	17,650, 3611, 1468
*R*_int_	0.145
(sin θ/λ)_max_ (Å^−1^)	0.594
Refinement	
$R\left[ {F^{ 2} > { 2}\sigma \left( {F^{ 2} } \right)} \right]$, *wR*(*F*^2^), *S*	0.071, 0.184, 1.00
No. of reflections	3611
No. of parameters	266
H-atom treatment	H-atom parameters constrained
Δρ_max_, Δρ_min_ (e Å^−3^)	0.12, − 0.14

## Experimental

### Chemistry

#### General

Melting points were determined on Electrothermal IA 9100 capillary melting point apparatus and are uncorrected. UV spectra were recorded on UV–Vis spectrophotometer model type of Genesys 10 s and expressed in nm. Thermo Scientific. Glass cuvettes were used. All the samples were dissolve in chloroform or methanol. FT-IR spectroscopic studies were carried out on FTIR spectrophotometer 1000 model Perkin Elmer at room temperature 25 °C. KBr pellets were dried in oven and scanned for calibration purpose. ^1^H NMR spectra of compounds were recorded on a Bruker Ascend TM 600 MHz machine, while the spectra of compounds **12**, **16**, **17** were recorded on 500 MHz NMR spectrometers. The chemical shifts (δ) are presented with references to CDCl_3_ (δ: 7.25) and TMS (*δ*: 0.00) as the internal reference. Electron-spray ionization mass spectra in positive mode (ESI–MS) were recorded on a Bruker Esquire 3000 spectrometer. Column chromatography purifications were carried out on Silica Gel 60 (Merck, 70–230 mesh, ASTM) and flash silica gel (230–400 mesh, ASTM, Merck). The purity of all compounds were checked by thin-layer chromatography (TLC) and ^1^H-NMR spectra. All reagents used were of analytical grade. All the chemicals were purchased from Aldrich, U.S.A. Other reagents were purchased from Sinopharm Chemical Reagent Co. Ltd., China.

### Synthetic procedures

#### Method A (acid-catalyzed)

A typical Claisen-Schmidt condensation reaction procedure was used to prepare all curuminoids. Appropriate mono ketone (cyclohexanone, acetone and cyclopentanone) 10 mol (1 equiv) was dissolved in absolute ethanol (15–20 mL). Substituted benzaldehydes 20 mol, (2 equiv) was added slowly. About 1–2 mL concentrated HCl was added drop wise over 5–10 min in a stirred mixture of ketone. The reaction mixture was stirred overnight (12–24 h). The product was monitored by comparing the Co-TLC with the starting material. The products were extracted with ethyl acetate by dissolving the compounds in distilled water (100 mL). Curcuminoids were purified by silica gel column chromatography (ethyl acetate/hexane) and re-crystallized with hot solution of ethyl acetate and ethanol.

#### Method B (base-catalyzed)

The general procedure Claisen–Schmidt condensation reaction was used to synthesize curcuminoids by using this method involved in addition of certain amount of mono ketone (cyclohexanone, acetone and cyclopentanone) to a solution of substituted aldehydes in MeOH or C_2_H_5_OH by adding KOH or NaOH. The reaction mixture is stirred at room temperature and monitored by TLC. The products are extracted and purified as described as in acid catalysed [43, 44].

##### (2*E*,6*E*)-2,6-bis(2-Methoxybenzylidene)cyclohexanone (**1**)

Yellow liquid; yield (86%); UV–Vis (CHCl_3_) λ_max_: 302, 339 nm; IR (KBr,) v 3023 (Ar C–H stretch), 1636 (C=O), 1527 (Ar C=C<) cm^−1^; ^1^H NMR (CDCl_3_, 600 MHz) δ 1.75 (m, 2H, 4-H), 2.84 (m, 4H, 3, 5-H), 3.86 (s, 6H, OCH_3_, C-2′ & C-2″), 6.92 (m, 2H, 3′, 3″-H), 6.96 (m, 2H, 5′, 5″-H), 7.32 (m, 2H, 4′, 4″-H), 7.33–7.30 (m, 4H, 4′, 4″, 6′, 6″-H), 7.98 (brs, 2H, –C=C–H). ^13^C NMR (CDCl_3_, 150 MHz) δ 23.5 (C-4), 28.6 (C-3, C-5), 55.5 (OCH_3_), 110.6 (C-3′, C-3″), 119.9 (C-5′, C-5″), 125.2 (C-4′, C-4″, C-6′, C-6″), 130.3 (C-1′, C-1″), 132.5 (C-2, C-6), 136.6 (–C=C–H), 158.4 (C-2′, C-2″), 190.6 (C=O); EI-MS *m/z* 334.0 (10), 303.1 (20), 240.3 (14), 161.2 (19), 107.4 (23), 77.0 (64); HREI-MS for C_22_H_22_O_3_ M^+^, calcd.: *m/z* 334.1575, found: *m/z* 334.1589.

##### (2*E*,6*E)*-2,6-bis(4-Methoxybenzylidene)cyclohexanone (**2**)

Yellow crystals; yield (74%); m.p. 152–153 °C (lit. [29] 148–149 °C); UV–Vis (CHCl_3_) λ_max_: 362 nm; IR (KBr) v 3010 (Ar C–H stretch), 1660 (C=O), 1508–1594 (Ar C=C) cm^−1^; ^1^H NMR (CDCl_3_, 600 MHz) δ 1.80 (m, 2H, 4-H), 2.92 (m, 4H, 3, 5-H), 3.84 (s, 6H, OCH_3_, C-4′, 4″), 6.93 (d, 4H, 3′, 3″, 5′, 5″-H, *J *= 6.78 Hz), 7.45 (d, 4H, 2′, 2″, 6′, 6″-H, *J *= 6.78 Hz), 7.76 (brs, 2H, –C=C–H); EI-MS *m/z* 334.0 (100), 303.45 (36), 240.1 (23), 161.2 (10), 107.0 (28); HREI-MS for C_22_H_22_O_3_ M^+^, calcd.: *m/z* 334.1568, found: *m/z* 334.1573.

##### (2*E*,6*E*)-2,6-bis(2,3-Dimethoxybenzylidene)cyclohexanone (**3**)

Yellow crystals; yield (92%); m.p. 105–106 °C (lit. [36] 107–109 °C); UV–Vis (CHCl_3_) λ_max_: 324 nm; IR (KBr) v 3023 (Ar C–H stretch), 1622 (C=O), 1536–1536 (Ar C=C) cm^−1^; ^1^H NMR (CDCl_3_, 600 MHz) δ 1.75 (m, 2H, 4-H), 2.80 (m, 4H, 3, 5-H), 3.82 (s, 6H, OCH_3_, C3′, 3″), 3.88 (s, 6H, OCH_3_, C-2′, 2″), 6.93 (m, 4H, 4′, 4″, 6′, 6″-H), 7.06 (brt, 2H, 5′, 5″-H, *J *= 7.98 Hz), 7.94 (brs, 2H, –C=C–H); ^13^C NMR, (150 MHz, CDCI_3_) δ 23.3 (C-4), 28.78 (C-3, C-5), 55.9 (OCH_3_), 61.2 (OCH_3_), 112.8 (C-5′, C-5″), 122.2 (C-4′, C-4″), 123.5 (C-6′, C-6″), 130.5 (C-1′, C-1″), 132.5 (C-2, C-6), 137.5 (C=C–H), 152.9 (C-2′, C-2″, C-3′, C-3″), 190.4 (C=O); EI-MS *m/z* 394 (5), 363.0 (100), 331.2 (68), 161.23 (86), 227.33 (24), 136.18 (29); HREI-MS for C_24_H_26_O_5_ M^+^, calcd.: *m/z* 394.1783, found: *m/z* 394.1778.

##### (2*E*,6*E*)-2,6-bis(4-Hydroxy-3-methoxybenzylidene)cyclohexanone (**5**)

Synthesis, purification and experimental data of compound **5** was recently published by us [31].

##### (2*E*,6*E*)-2,6-bis(2-Chlorobenzylidene)cyclohexanone (**6**)

Yellow crystals; yield (68%); m.p. 109–110 °C (lit. [36] 94–95 °C); UV–Vis (CHCl_3_) λ_max_: 320 nm; IR (KBr) v 3073 (Ar C–H stretch), 1663 (C=O), 1574–1433 (Ar C=C) cm^−1^; ^1^H NMR (CDCl_3_, 600 MHz) δ 1.76 (m, 2H, 4-H), 2.78 (m, 4H, 3, 5-H), 7.33 (m, 2H, 3′, 3″-H), 7.28 (m, 4H, 4′, 4″, 5′, 5″-H), 7.44 (m, 2H, 6′, 6″-H), 7.91 (brs, 2H, –C=C–H); EI-MS *m/z* 343.0 (5), 307 (100), 272 (8), 166 (4), 138 (6), 112 (17); HREI-MS for C_20_H_16_Cl_2_O M^+^, calcd.: *m/z* 342.0578, found: *m/z* 342.0572.

##### (2*E*,6*E*)-2,6-bis(4-Chlorobenzylidene)cyclohexanone (**7**)

Yellow crystals; yield (86%); m.p. 149–153 °C (lit. [29] 147–149 °C); UV–Vis (CHCl_3_) λ_max_: 335 nm; IR (KBr) v 3063 (Ar C–H stretch), 1604 (C=O), 1576–1487 (Ar C=C) cm^−1^; ^1^H NMR (CDCI_3_, 500 MHz) δ 1.80 (m, 2H, 4-H), 2.89 (m, 4H, 3, 5-H), 7.34 (m, 2H, 2′, 2″-H), 7.34 (m, 2H, 3′, 3″-H), 7.34 (m, 2H, 5′, 5″-H), 7.34 (m, 2H, 6′, 6″-H), 7.73 (brs, 2H, –C=C–H); EI-MS *m/z* 343 (76), 307 (87), 272 (71), 244 (31), 166 (14), 138 (22), 112 (9); HREI-MS for C_20_H_16_Cl_2_O M^+^, calcd.: *m/z* 342.0678, found: *m/z* 342.0672.

##### (2*E*,6*E*)-2,6-bis(3,4-Dimethoxybenzylidene)cyclohexanone (**10**)

Yellow crystals; yield (74%); m.p. 146–149 °C (lit. [37] 148–150 °C); UV–Vis (CHCl_3_) λ_max_: 373 nm; IR (KBr) v 3036 (Ar C–H stretch), 1614 (C=O), 1489–1462 (Ar C=C) cm^−1^; ^1^H NMR (CDCl_3_, 600 MHz) δ 1.83 (m, 2H, 4-H), 2.95 (m, 4H, 3, 5-H), 3.90 (s, 6H, OCH_3_, C-3′, 3″), 3.92 (s, 6H, OCH_3_, C-4′, 4″), 6.91 (d, 2H, 5′, 5″-H, *J *= 8.34 Hz), 7.02 (d, 2H, 2′, 2″-H, *J *= 1.92 Hz), 7.12 (dd, 2H, 6′, 6″-H, *J *= 8.34, 1.92 Hz), 7.76 (brs, 2H, –C=C–H); EI-MS *m/z* 394 (3), 363 (100), 331 (9), 161 (4), 227 (23), 136 (3), 77 (31); HREI-MS for C_24_H_26_O_5_ M^+^, calcd.: *m/z* 394.1784, found: *m/z* 394.1787.

##### (1*E*,4*E*)-1,5-bis(2-Methoxyphenyl)-penta-1,4-dien-3-one (**11**)

Yellow crystals; yield (66%); m.p. 111–114 °C (lit. [45] 118–120 °C); UV–Vis (CHCl_3_)λ_max_: 312, 360 nm; IR (KBr) v 3023 (Ar C–H stretch), 1614 (C=O), 1489–1462 (Ar C=C) cm^−1^; ^1^H NMR (CDCl_3_, 600 MHz) δ 3.90 (s, 6H, OCH_3_, C-2′, 2″), 6.93 (d, 2H, 3′, 3″-H, *J *= 8.34 Hz), 6.99 (t, 2H, 4′, 4″-H, *J *= 8.46, 7.4 Hz), 7.18 (d, 2H, 2, 4-H, *J *= 16.08 Hz), 7.36 (td, 2H, 5′, 5″-H, *J *= 7.4, 1.6 Hz), 7.62 (dd, 2H, 6′, 6″-H, *J *= 7.6, 1.6 Hz), 8.08 (d, 2H, 1, 5-H, *J *= 16.08 Hz); EI-MS *m/z* 294 (100), 263 (8), 234 (15), 186 (50), 161 (36), 133 (33), 77 (16); HREI-MS for C_19_H_18_O_3_ M^+^, calcd.: *m/z* 294.1255, found: *m/z* 294.1251.

##### (1*E*,4*E*)-1,5-bis(4-Methoxyphenyl)-penta-1,4-dien-3-one (**12**)

Yellow crystals; yield (79%); m.p. 121–122 °C (lit. [46] 119–120 °C); UV–Vis (CHCl_3_) λ_max_: 354 nm; IR (KBr) v 3033 (Ar C–H stretch), 1624 (C=O), 1590–1488 (Ar C=C) cm^−1^. ^1^H NMR (CDCl_3_, 500 MHz) δ 3.87 (s, 6H, OCH_3_, C-4′, 4″), 6.94 (d, 4H, 3′, 3″, 5′, 5″-H, *J *= 8.75 Hz), 6.99 (d, 2H, 2, 4-H, *J *= 16.0 Hz), 7.60 (d, 4H, 2′, 2″, 6′, 6″-H, *J *= 8.75 Hz), 7.74 (d, 2H, 1, 5-H, *J *= 16.0 Hz); EI-MS *m/z* 294.14 (100), 263 (15), 234 (20), 186 (54), 161 (38), 133 (36), 77 (21); HREI-MS for C_19_H_18_O_3_ M^+^, calcd.: *m/z* 294.1264, found: *m/z* 294.1257.

##### (1*E*,4*E*)-1,5-bis(2,3-Dimethoxyphenyl)-penta-1,4-dien-3-one (**13**)

Yellow solid; yield (68%); m.p. 103–104 °C (lit. [36] 106–108 °C); UV–Vis (CHCl_3_) λ_max_: 330 nm; IR (KBr) v 3011–2943 (Ar C–H stretch), 1619 (C=O), 1577–1479 (Ar C=C) cm^−1^; ^1^H NMR (CDCl_3_, 600 MHz) δ 3.87 (s, 12H, OCH_3_, C-2′, 2″, 3′, 3″), 6.97 (dd, 2H, 4′, 4″-H, *J* = 8.16, 1.44 Hz), 7.10 (t, 2H, 5′, 5″-H, *J* = 8.04, 8.00 Hz), 7.16 (d, 2H, 2, 4-H, *J* = 16.1 Hz), 7.26 (dd, 2H, 6′, 6″-H, *J* = 8.00, 1.44 Hz), 8.04 (d, 2H, 1, 5-H, *J* = 16.1 Hz); EI-MS *m/z* 354 (5), 323 (3), 230 (4), 186 (9), 132 (13), 191 (4), 163 (7), 77 (52); HREI-MS for C_21_H_22_O_5_ M^+^, calcd.: *m/z* 354.1467, found: *m/z* 394.1462.

##### (1*E*,4*E*)-1,5-bis(4-Chlorophenyl)-penta-1,4-dien-3-one (**16**)

Yellow solid; yield (72%); m.p. 193–195 °C (lit. [36] 192–193 °C); UV–Vis (CHCl_3_) λ_max_: 333 nm; IR (KBr) v 3065 (Ar C–H stretch), 1608 (C=O), 1584–1489 (Ar C=C str.) cm^−1^; ^1^H-NMR (CDCl_3_, 500 MHz) δ 7.04 (d, 2H, 2, 4-H, *J *= 15.9 Hz), 7.40 (dd, 4H, 3′, 3″, 5′, 5″-H, *J *= 8.60 Hz), 7.56 (d, 4H, 2′, 2″, 6′, 6″-H, *J *= 8.60 Hz), 7.70 (d, H, 1, 5-H, *J *= 15.9 Hz); ^13^C NMR (150 MHz, CDCl_3_) δ 126.0 (C-2, 4), 128.7 (C-3′, 3″), 128.7 (C-5′, 5″), 129.3 (C-2′, 2″), 129.3 (C-6′, 6″), 133.3 (C-1′, 1″), 136.5 (C-4′, 4″), 142.1 (C-1, 5), 188.3 (C=O); EI-MS *m/z* 302 (60), 267 (32), 232 (5), 203 (20), 165 (35), 137 (49), 77 (5); HREI-MS for C_17_H_12_Cl_2_O M^+^, calcd.: *m/z* 302.0265, found: *m/z* 302.0259.

##### (1*E*,4*E*)-1,5-bis(2,4,6-Trimethoxyphenyl)-penta-1,4-dien-3-one (**17**)

Yellow solid; yield (68%); m.p. 213–215 °C (lit. [36] 209–211 °C); UV–Vis (CHCl_3_) λ_max_: 381 nm. IR (KBr) 3002 (Ar C–H str.), 1629 (C=O), 1561–1466 (Ar C=C) cm^−1^; ^1^H NMR (CDCl_3_, 600 MHz) δ 3.74 (s, 6H, 2 × OCH_3_, C-4′, 4″), 3.85 (s, 12H, 4 × OCH_3_, C-2′, 2″, 6′, 6″), 6.13 (brs, 4H, 3′, 3″, 5′, 5″-H), 7.46 (d, 2H, 2, 4-H, *J *= 16.30 Hz), 8.12 (d, 2H, 1, 5-H, *J *= 16.30 Hz); EI-MS *m/z* 414 (5), 131 (6), 105 (10); HREI-MS for C_23_H_26_O_7_ M^+^, calcd.: *m/z* 414.1671, found: *m/z* 414.1679.

##### (2*E*,5*E*)-2,5-bis(4-Methoxybenzylidene)cyclopentanone (**19**)

Yellow solid; yield (66%); m.p. 150–155.5 °C (lit. [29] 158–161 °C); UV–Vis (CHCl_3_) λ_max_: 391 nm; IR (KBr) v 2964 (Ar C–H stretch), 1696 (C=O), 1597–1509 (Ar C=C) cm^−1^; ^1^H NMR (CDCI_3_, 600 MHz) δ 3.09 (brs, 4H, 3, 4-H), 3.86 (s, 6H, 2 × OCH_3_, C-4′, 4″), 6.98 (brd, 2H, 5′, 5″-H, *J *= 8.34 Hz), 6.98 (brd, 2H, 3′, 3″-H, *J *= 8.34 Hz), 7.57 (brt, 2H, 2′, 2″-H, *J *= 8.52 Hz), 7.57 (brt, 2H, 6′, 6″-H, *J *= 8.52 Hz), 7.58 (brs, 2H, –C=C–H); EI-MS *m/z* 320 (11), 213 (8), 183 (5), 131 (12), 77 (16); HREI-MS for C_21_H_20_O_3_ M^+^, calcd.: *m/z* 320.1412, found: *m/z* 320.140.

##### (2*E*,5*E*)-2,5-bis(2,3-Dimethoxybenzylidene)cyclopentanone (**20**)

Yellow solid; yield (54%); m.p. 156–158 °C (lit. [36] 155–157 °C); UV–Vis (CHCl_3_) λ_max_: 346 nm; IR (KBr) v 3032 (Ar C-H stretch), 1694 (C=C), 1622 (C=O), 1584–1489 (Ar C=C) cm^−1^; ^1^H NMR (CDCI_3_, 600 MHz) δ 3.02 (brs, 4H, 3, 4-H), 3.87 (s, 6H, OCH_3_, C-2′, 2″), 3.88 (s, 6H, OCH_3_, C-3′, 3″), 6.96 (m, 2H, 4′, 4″-H), 7.10 (t, 2H, 5′, 5″-H, *J *= 7.9 Hz), 7.16 (dd, 2H, 6′, 6″-H, *J *= 7.9 Hz), 7.93 (brs, 2H, –C=C–H); EI-MS *m/z* 380 (3), 349 (4), 163 (10), 137 (10), 98 (18); HREI-MS for C_23_H_24_O_5_ M^+^, calcd.: *m/z* 380.1618, found: *m/z* 380.1623.

##### (2*E*,5*E*)-2,5-bis(4-Hydroxy-3-methoxybenzylidene)cyclopentanone (**22**)

Yellow solid; yield (58%); m.p. 212–214 °C (lit. [47] 214 °C); UV–Vis (CHCl_3_) λ_max_: 388 nm; IR (KBr) v 3043 (Ar C–H stretch), 1690 (C=C), 1620 (C=O), 1588–1485 (Ar C=C) cm^−1^; ^1^H NMR (CDCl_3_, 500 MHz) δ 3.03 (s, 4H, 3, 4-H), 3.88 (s, 6H, OCH_3_, C-3′, 3″), 6.92 (d, 2H, 5′, 5″-H, *J *= 8.30 Hz), 7.04 (brs, 2H, 2′, 2″-H), 7.14 (dd, 2H, 5′, 5″-H, *J *= 8.30, 1.65 Hz), 7.46 (brs, 2H, –C=C–H); EI-MS *m/*z 352; HREI-MS for C_21_H_20_O_5_ M^+^, calcd.: *m/z* 352.1310, found: *m/z* 352.1305.

##### (2*E*,5*E*)-2,5-bis(3,4-Dimethoxybenzylidene)cyclopentanone (**23**)

Yellow solid; yield (54%); m.p. 191–193 °C (lit. [37] 188–190 °C); UV–Vis (CHCl_3_) λ_max_: 368 nm; IR (KBr) v 3006 (Ar C–H stretch), 1693 (C=O), 1592–1515 (Ar C=C) cm^−1^; ^1^H NMR (CDCl_3_, 600 MHz) δ 3.12 (brs, 4H, 3, 4-H), 3.94, 3.93 (s, 12H, 4 × OCH_3_, C-3′, 3″, 4′, 4″), 6.96 (d, 2H, 5′, 5″-H, *J *= 8.34 Hz), 7.14 (s, 2H, 2′, 2″-H), 7.24 (dd, 2H, 6′, 6″-H, *J *= 8.34 Hz), 7.55 (brs, 2H, –C=C–H); ^13^C NMR (150 MHz, CDCl_3_) δ 26.3 (C-3, 4), 56.0 (C–O), 111.2 (C-2′, 2″), 113.5 (C-5′, 5″), 124.6 (C-6′, 6″), 129.0 (C-1′, 1″), 133.7 (–C=C–H), 148.9 (C-2, 5), 150.3 (C-3′, 3″), 150.3 (C-4′, 4″), 196.0 (C=O); EI-MS *m/z* 380.1 (5), 191.0 (10), 132.2 (18), 77.2 (55); HREI-MS for C_23_H_24_O_5_ M^+^, calcd.: *m/z* 380.1624, found: *m/z* 380.1619.

### Anticancer activity

#### Sample preparation

Stock samples at 1 mg/mL of dimethyl sulfoxide (DMSO) (Sigma-Aldrich, USA) were prepared and keep at 4 °C.

### MTT cell viability assay

Breast cancer MCF-7 and MDA-MB-231 cells, chronic myelogenous leukemia K562 cells, and cervical cancer HeLa cells lines were purchased from American Type Culture Collection (ATCC, USA) and cultured at 37 °C, 5% CO_2_ and 90% humidity using RPMI-1640 medium (Sigma-Aldrich, USA) supplemented with 10% Foetal Bovine Serum (FBS) (Thermo Fisher Scientific, USA). For MTT (3-(4,5-dimethylthiazolyl-2)-2,5-diphenyltetrazolium bromide) cell viability assay [48], MCF-7, MDA-MB-231, K562 and HeLa cells were seeded overnight in 96-well plates at 8 × 10^4^ cells/well at 37 °C of CO_2_ [49]. Then, 100 µL of media was discarded for all well-plates and compounds were serially diluted into the seeded cells at the concentration ranging between 30–0.47 µg/mL with cells treated with 3% DMSO (Sigma-Aldrich, USA) as the negative control. All samples were tested for triplicates. After 72 h of incubation, all well was added with 20 µL of MTT solution (5 mg/mL) and further incubated for 3 h. At that point, 170 µL of solution were discarded and 100 µL of DMSO (Sigma-Aldrich, USA) was added to all wells. Finally, absorbance was recorded by ELISA plate reader (Biotek-Instruments, USA) at the wavelength of 570 nm. Percentage of cell viability was calculated using following formula [38, 39]. The assay was performed in triplicate to calculate the half maximal inhibitory concentration (IC_50_) values. Doxorubicin was used as a positive control.

Cell viability (%) = [OD sample at 570 nm/OD negative control at 570 nm] × 100%

IC_50_ value (concentration of compounds inhibited 50% of cell viability) was determined from the graph of cell viability vs absorbance.

### X-ray crystallographic analysis

X-ray analysis for all these samples were performed using Bruker APEX II DUO CCD diffractometer, employing MoKα radiation (λ = 0.71073 Å) with φ and ω scans, at room temperature. Data reduction and absorption correction were performed using SAINT and SADABS programs [50–53]. The structures of compound **4** was solved by direct methods and refined by full-matrix least-squares techniques on *F*^2^ using SHELXTL software package. Crystallographic data of the reported structures have been deposited at the Cambridge Crystallographic Data Centre with CCDC deposition numbers of 1548735. Copy of available material can be obtained free of charge, on application to CCDC, 12 Union Road, Cambridge CB2 1EZ, UK, (Fax: +44-(0)1223-336033 or e-mail: deposit@ccdc.cam.ac.uk).

## Conclusions

In conclusion, we have examined three series of curcumin analogues against four types (HeLa, K562, MCF-7 and MDA-MB-231) cancer cell lines. Curcuminoids with diferuloyl (4-hydroxy-3-methoxycinnamoyl) moiety with mono carbonyl exhibiting potential cytotoxic properties. The compound **14** was exhibited (IC_50_ = 3.02 ± 1.20 and 1.52 ± 0.60 µg/mL) against MCF-7 and MDA-MB-231 breast cancer cell lines. Structure activity relationship revealed that the role of methoxy groups are important. Curcumin derivatives, **4**, **5**, **9**, **14**, **11** and **17** exhibited significant cytotoxic activity (Table 1). Curcuminoids with acetone series such as 2,5-dimethoxy substituted with mono ketones were found to be more selective and potential cytotoxic agents, which could be the best templet for future drug discovery against selective cancer especially breast cancer lines.

## Abbreviations

HeLa: Henrietta Lacks
MCF-7: Michigan Cancer Foundation-7
HCl: hydrochloric acid
TMS: tetramethylsilane
CDCI_3_: chloroform
TLC: thin layer chromatography
MeOH: methanol
EtOH: ethanol
KOH: potassium hydroxide
NaOH: sodium hydroxide
NMR: nuclear magnetic resonance
IR: infrared radiation
UV-Vis: ultraviolet visible
MS: mass spectrometry
DMSO: dimethyl sulfoxide
MTT: (3-(4,5-dimethylthiazolyl-2)-2,5-diphenyltetrazolium bromide)
